# COVID-19 and associations with frailty and multimorbidity: a prospective analysis of UK Biobank participants

**DOI:** 10.1093/geroni/igaa057.3435

**Published:** 2020-12-16

**Authors:** Stephen Woolford, Stefania D’Angelo, Elizabeth Curtis, Camille Parsons, Elaine Dennison, Harnish Patel, Cyrus Cooper, Nicholas Harvey

**Affiliations:** 1 MRC Lifecourse Epidemiology Unit, Southampton, England, United Kingdom; 2 University Hospital Southampton NHS Foundation Trust, Southampton, England, United Kingdom

## Abstract

Frailty and multimorbidity, which are more prevalent in older people, have been suggested as risk factors for severe COVID-19 disease. We investigated whether frailty and multimorbidity were associated with risk of hospitalisation with COVID-19 in the UK Biobank. 502,640 participants aged 40-69 years at baseline (54-79 years at COVID-19 testing) were recruited across UK 2006-10. A modified assessment of frailty using Fried’s classification was generated from baseline data. COVID-19 test results (England) were available 16/03/2020-01/06/2020, mostly taken in hospital settings. Logistic regression was used to discern associations between frailty, multimorbidity and COVID-19 diagnoses, adjusting for sex, age, BMI, ethnicity, education, smoking and number of comorbidity groupings, comparing COVID-19 positive, COVID-19 negative and non-tested groups. 4,510 participants were tested for COVID-19 (positive=1,326, negative=3,184). 497,996 participants were not tested. Compared to the non-tested group, after adjustment, COVID-19 positive participants were more likely to be frail (OR=1.4 [95%CI=1.1, 1.8]), report slow walking speed (OR=1.3 [1.1, 1.6]), report two or more falls in the past year (OR=1.3 [1.0, 1.5]) and be multimorbid (≥4 comorbidity groupings vs 0-1: OR=1.9 [1.5, 2.3]). However, similar strength of associations were apparent when comparing COVID-19 negative and non-tested groups. Furthermore, frailty and multimorbidity were not associated with COVID-19 diagnoses, when comparing COVID-19 positive and COVID-19 negative participants. In conclusion, frailty and multimorbidity do not appear to aid risk stratification, in terms of a positive versus negative results of COVID-19 testing. Investigation of the prognostic value of these markers for adverse clinical sequelae following COVID-19 disease is urgently needed.

